# Supplementary material for: Perspectives on dental health and oral hygiene practice from US adolescents and young adults during the COVID-19 pandemic
Source: PLoS One. 2023 Jan 19;18(1):e0280533. doi: 10.1371/journal.pone.0280533 (PMC9851495; doi:10.1371/journal.pone.0280533)
Supplement: Attachment — Submitted filename: PlosOne_Response_Ltr_11222.docx [file pone.0280533.s001.docx]

**DEPARTMENT OF FAMILY MEDICINE**

2800 Plymouth Road

Building 14- Room G128

Ann Arbor, Michigan 48109-2800

734 998-7120 Research Office

734 998-7335 Fax

November 21, 2022

Dear Dr. Chaubal,

Thank you for your email dated October 24, 2022, regarding our manuscript. We greatly appreciate the suggestions and have edited our manuscript accordingly. Our point-by-point responses to the comments are detailed below. Thank you for considering our revised manuscript for PLOS ONE.

Sincerely,

Tammy Chang, MD, MPH, MS

Associate Professor

University of Michigan

**RESPONSE TO REVIEWERS**

Reviewer #1: The manuscript discusses the oral health awareness of a very important age group. The questions asked could have included the diet of the individuals and if they had access to a dentist easily. Also, the socioeconomic status is not mentioned in the demographics. The sample size is also inadequate to make a general observation of all adolescents and young adults. The questions on COVID-19 impact lack depth and could have added information if the oral health status like bleeding gums were exacerbated at that time. Overall, not a very insightful study. Adding a larger sample size with relevant questions like the above will help us get a true picture of this population.

**RESPONSE**: The following statements have been added to the limitations section to address this reviewer's concerns:

Participants represent a large nationwide sample of youth that opted in to a text messaging poll.  However, the sample is not nationally representative, which limits the generalizeability of our findings. The responses were also taken in May 2021, a very dynamic time where thoughts and opinions of COVID-19 in responses may change from the start of the pandemic to today. MyVoice is an open-ended poll designed to be only 5 questions to minimize burden on our youth participants, though this limits our ability to probe more deeply into the experiences of youth.

Reviewer #2: line 168 - in the sentence the surveyed and perceived should be separated by a comma(,)
line 170- there is extra comma after the hygiene word, please remove.
check and go through similarly such commas are being added, please remove those also

**RESPONSE**:  The placement of the commas noted appears appropriate, though we will follow the guidance of the copy editor if changes are needed.

**EDITORS COMMENTS**

When submitting your revision, we need you to address these additional requirements.

1. Please ensure that your manuscript meets PLOS ONE's style requirements, including those for file naming. The PLOS ONE style templates can be found at 
https://journals.plos.org/plosone/s/file?id=wjVg/PLOSOne_formatting_sample_main_body.pdf and 
<https://journals.plos.org/plosone/s/file?id=ba62/PLOSOne_formatting_sample_title_authors_affiliations.pdf>

**RESPONSE**: We have added the formatting requirements that were overlooked.  .

2. Thank you for stating the following in the Acknowledgments Section of your manuscript:

"This research was funded by the Michigan Institute for Clinical & Health Research and the University of Michigan Department of Family Medicine. Funders had no part in the study design, data collection, analyses, interpretation of findings, or the decision to submit the manuscript for publication."

We note that you have provided funding information that is not currently declared in your Funding Statement. However, funding information should not appear in the Acknowledgments section or other areas of your manuscript. We will only publish funding information present in the Funding Statement section of the online submission form.

Please remove any funding-related text from the manuscript and let us know how you would like to update your Funding Statement. Currently, your Funding Statement reads as follows:

"TC received funding for this research from the Michigan Institute for Clinical & Health Research and the University of Michigan Department of Family Medicine. The funders had no role in study design, data collection and analysis, decision to publish, or preparation of the manuscript."

Please include your amended statements within your cover letter; we will change the online submission form on your behalf.

**RESPONSE**:  The above funding statement is correct and no amendment is needed.  Thank you for placing it in the Funding Statement section.

3. We note that you have indicated that data from this study are available upon request. PLOS only allows data to be available upon request if there are legal or ethical restrictions on sharing data publicly. For more information on unacceptable data access restrictions, please see http://journals.plos.org/plosone/s/data-availability#loc-unacceptable-data-access-restrictions.

In your revised cover letter, please address the following prompts:

a) If there are ethical or legal restrictions on sharing a de-identified data set, please explain them in detail (e.g., data contain potentially sensitive information, data are owned by a third-party organization, etc.) and who has imposed them (e.g., an ethics committee). Please also provide contact information for a data access committee, ethics committee, or other institutional body to which data requests may be sent.

b) If there are no restrictions, please upload the minimal anonymized data set necessary to replicate your study findings as either Supporting Information files or to a stable, public repository and provide us with the relevant URLs, DOIs, or accession numbers. For a list of acceptable repositories, please see http://journals.plos.org/plosone/s/data-availability#loc-recommended-repositories.

We will update your Data Availability statement on your behalf to reflect the information you provide.

**RESPONSE**:  As part of our NIH Certificate of Confidentiality, even de-identified open-ended responses cannot be shared publicly without agreement to the terms of data protection for the use of such data.  This is because responses may be de-identified of traditional identifiers (demographics, location, etc), however, the youth's responses themselves may include identifying information.  If data is requested, the requestor can sign a data use agreement that ensures protection of our participants to receive the data.
